# Early Outcomes After Discontinuing Routine Gentamicin Irrigation During AMS 800 Artificial Urinary Sphincter Implantation: A Single-Centre Retrospective Before–After Cohort Study

**DOI:** 10.3390/antibiotics15090894

**Published:** 2026-09-11

**Authors:** Michał Andrzej Skrzypczyk, Anna Pliszka, Łukasz Białek, Marta Rydzińska, Wojciech Karoń, Jakub Dobruch, Artur Lemiński

**Affiliations:** 1 Department of Urology, Centre of Postgraduate Medical Education, 00-416 Warsaw, Poland; 2 Department of General and Oncological Urology, Independent Public Provincial Hospital in Szczecin, 71-455 Szczecin, Poland; 3 Department of Biochemical Sciences, Pomeranian Medical University, 71-460 Szczecin, Poland

**Keywords:** artificial urinary sphincter, AMS 800, gentamicin, antibiotic irrigation, surgical-site infection, urethral erosion, device explantation, antimicrobial stewardship, before–after study

## Abstract

**Background/Objectives**: Routine local gentamicin irrigation has been used during artificial urinary sphincter (AUS) implantation despite limited procedure-specific evidence. We evaluated early outcomes after its calendar-defined discontinuation. **Methods**: This single-centre retrospective before–after cohort included 173 uncoated AMS 800 procedures in 164 men: 59 under a gentamicin-containing local protocol and 114 after gentamicin withdrawal. Saline device immersion and Betadine-containing intraoperative irrigation of the operative field and both wounds continued in both periods. Clinician-recorded surgical-site infection (SSI) within 90 days was the antimicrobial outcome of greatest direct relevance. Complete explantation within 90 days for SSI and/or urethral erosion was an exploratory device-related endpoint; all-cause complications at 30 and 90 days were secondary safety outcomes. **Results**: SSI occurred after 1/59 (1.7%) gentamicin-protocol and 2/114 (1.8%) gentamicin-free procedures (risk difference +0.1 percentage points, 95% confidence interval −7.4 to +4.7; *p* = 1.000). Erosion occurred after 0/59 and 4/114 (3.5%) procedures; all four led to complete explantation. The explantation endpoint occurred after 1/59 versus 4/114 procedures (risk difference +1.8 percentage points, 95% confidence interval −5.8 to +7.2; *p* = 0.662). Thirty-day all-cause complications occurred after 14/59 versus 16/114 procedures and 90-day complications after 15/59 versus 26/114. **Conclusions**: No statistically significant between-period difference was detected in SSI, exploratory device-related explantation, or broad all-cause complications. Sparse events, wide confidence intervals and calendar-defined exposure amid concurrent era changes preclude conclusions regarding equivalence or absence of clinically relevant benefit or harm.

## 1. Introduction

Artificial urinary sphincter (AUS) implantation remains a reference surgical option for moderate-to-severe male stress urinary incontinence, particularly after radical prostatectomy. Although the AMS 800 device (Boston Scientific Corporation, Marlborough, MA, USA) has a long history of clinical use and well-established functional efficacy, infection, erosion, device malfunction and early postoperative morbidity remain clinically important because they may lead to reoperation, device explantation and delayed recovery [1].

Infection prevention in prosthetic urology combines preoperative urine assessment, appropriately timed systemic antimicrobial prophylaxis, meticulous surgical technique and strategies intended to reduce bacterial inoculation. Local antibiotic irrigation of operative wounds and prosthetic components has historically been used in some centres, but this practice is largely based on local tradition or extrapolation from other implant procedures rather than on direct evidence in AUS surgery.

International surgical-site infection guidance distinguishes systemic prophylaxis from local antimicrobial use. The World Health Organization (WHO) recommends against antibiotic incisional wound irrigation, while the Centers for Disease Control and Prevention identify antimicrobial soaking of prosthetic devices as an unresolved issue because of the absence of randomised evidence [2,3]. A recent network meta-analysis found greater support for antiseptic than antibiotic irrigation and emphasised antimicrobial-resistance concerns [4]. Surveys and stewardship discussions also show substantial practice variation and limited consensus regarding topical antibiotics [5,6].

Other local antimicrobial strategies have not established a clear additional benefit in AUS surgery. InhibiZone-coated AMS 800 devices have not consistently reduced infection or explantation compared with uncoated devices, and postoperative oral antibiotics have not been associated with lower explantation rates in nationwide observational data [7,8,9]. The American Urological Association Best Practice Statement addresses systemic antimicrobial prophylaxis for urological procedures but does not endorse routine local antibiotic irrigation as a standard step [10]. By contrast, the Asia-Pacific AMS 800 consensus statement recommends prophylactic antibiotics at the start of surgery and as irrigation (Grade B), particularly where InhibiZone-coated devices are unavailable, while acknowledging that conclusive evidence that antibiotic irrigation reduces prosthetic infection is lacking [11]. To our knowledge, no previous clinical study has directly compared a gentamicin-containing with a gentamicin-free local preparation protocol during AMS 800 implantation. At our centre, gentamicin was withdrawn from the local protocol after 1 February 2022, while device immersion in 0.9% sodium chloride and Betadine-containing intraoperative irrigation of the operative field and both wounds continued. We examined whether this change was followed by differences in early outcomes. Clinician-recorded SSI within 90 days was the outcome most directly relevant to the antimicrobial question. Complete explantation within 90 days for SSI and/or urethral erosion was a retrospectively defined exploratory device-related clinical endpoint rather than an antimicrobial efficacy endpoint because urethral erosion commonly has a noninfectious cause. Broad all-cause complications within 30 and 90 days were retained as secondary safety outcomes. The 90-day window was selected as a pragmatic period expected to capture most clinically apparent early events potentially related to intraoperative contamination; it does not exclude later presentation of an implantation-related infection. The question addressed is therefore the incremental value of local gentamicin within the prophylaxis protocol used at this centre, not the effects of discontinuing all local preparation measures or the safety of gentamicin withdrawal in settings using narrower or shorter antimicrobial regimens.

## 2. Materials and Methods

### 2.1. Study Design and Population

This retrospective single-centre before-after cohort study was reported in accordance with the Strengthening the Reporting of Observational Studies in Epidemiology (STROBE) framework [12]. Consecutive continence-prosthesis procedures performed between 12 June 2014 and 17 December 2025 were screened. The database was locked on 31 May 2026, after completion of the 90-day window for the final procedure. Of 262 screened procedures, 89 were excluded: 36 ZSI 375 implantations, 17 FlowSecure implantations, 3 Aroyo implantations, 7 Phorbas slings, 7 Adjustable Transobturator Male System (ATOMS) slings, 6 procedures in women, and 13 isolated component-only AMS 800 revisions for persistent urinary incontinence. The final cohort comprised 173 AMS 800 procedures in 164 patients. This study did not compare continence devices. Alternative devices were used more often during parts of the earlier period, and the database did not contain a prospectively standardised algorithm governing selection of AMS 800 rather than another continence prosthesis; temporal changes in AMS 800 candidacy and case mix were therefore considered potential era effects.

The procedure was the unit of analysis. Patient-level linkage identified 157 patients who contributed one procedure, five who contributed two and two who contributed three. The cohort included primary implantations, complete device exchanges and reimplantations after previous complete AUS explantation. The 13 isolated component-only procedures excluded during screening comprised cuff, pump or pressure-regulating balloon replacement without exchange of the complete system. Reimplantation after explantation was distinguished from complete device exchange during the same operation because it represents a clinically different reoperative setting.

All 173 procedures were performed by the same reconstructive urologist (M.A.S.) using the same core operative technique. During the study period, the surgeon performed approximately 150–200 reconstructive procedures annually, including 20–50 continence-prosthesis implantations, while AMS 800-specific volume and experience increased over time. Lower AMS 800 counts in some earlier years partly reflected greater use of alternative continence devices and do not represent low total prosthetic or reconstructive volume. The single-surgeon design removes between-surgeon variation but not temporal changes in experience, case mix, team organisation or device selection.

Variables extracted from the medical records included age, body mass index (BMI), cardiovascular disease, diabetes, antiplatelet and anticoagulant therapy, current smoking, radical prostatectomy, endoscopic surgery for benign prostatic hyperplasia, pelvic radiotherapy, antiandrogen therapy, prior endoscopic treatment for urethral stricture or vesicourethral anastomotic stenosis (VUAS), previous urethroplasty, type of AUS procedure (primary implantation, complete device exchange or reimplantation after previous complete explantation), standard versus transcorporal cuff placement, cuff size, pressure-regulating balloon range, operative time, intraoperative complications, length of stay, catheter duration and documented postoperative complications. Intraoperative complication was defined as any documented bladder or urethral injury, clinically relevant bleeding, or unexpected finding requiring additional management or modification of the planned dissection; it was recorded after 1/59 gentamicin-protocol procedures and 4/114 gentamicin-free procedures, with one missing value.

### 2.2. Exposure Definition and Perioperative Protocol

The exposure of interest was inclusion of gentamicin in the local device- and wound-preparation protocol. Through and including the procedure performed on 1 February 2022, the entire uncoated AMS 800 device was immersed during preparation in a solution made from five 80 mg/2 mL ampoules of gentamicin (total dose 400 mg) in 200 mL of 0.9% sodium chloride. The same gentamicin-containing solution was used to irrigate both operative wounds—the perineal wound and the lower-abdominal wound—before closure. In both periods, a Betadine-containing aqueous solution was also used intraoperatively to irrigate the operative field and both wounds during surgery, as described below. The exact gentamicin immersion duration and the volume allocated to the device and to each wound were not retained in the source records. The next procedure, performed on 2 February 2022, marked the start of the gentamicin-free period. From that date onward, the device continued to be immersed in 0.9% sodium chloride without gentamicin, Betadine-containing intraoperative irrigation of the operative field and both wounds continued, and gentamicin-containing device immersion and wound irrigation were discontinued. Discontinuation was a targeted removal of local gentamicin and was not part of a broader perioperative protocol revision at that date. Thus, the comparison evaluates withdrawal of gentamicin from an otherwise continuing local preparation pathway rather than irrigation versus no irrigation. The two periods are therefore termed the gentamicin protocol and the gentamicin-free protocol. The operative technique itself was unchanged at the discontinuation date.

All AMS 800 devices included in this cohort were uncoated and did not contain InhibiZone. Cuff placement was through a perineal incision; the pump and pressure-regulating balloon were placed through a separate lower-abdominal incision. A single cuff was used, either in a standard bulbar position or using a transcorporal approach when clinically indicated. The routine catheter pathway consisted of removal on postoperative day 1 and discharge after catheter removal when no complication was present.

Systemic perioperative prophylaxis was agreed with the hospital microbiologist and remained unchanged throughout the study. Intravenous ceftriaxone 1 g and amikacin 1 g, with amikacin dose adjustment according to estimated glomerular filtration rate when required, were administered approximately 15 min before the start of the operation. Intravenous metronidazole 500 mg was administered at 06:00, 14:00 and 22:00 on the operative day. All patients also received a 10-day oral post-discharge course: ciprofloxacin 500 mg twice daily through May 2023 and cefuroxime axetil 500 mg twice daily from June 2023. The change followed European regulatory restrictions and renewed warnings on systemic fluoroquinolone use after the European Medicines Agency safety review [13]. Accordingly, the cohort comprised three calendar-defined prophylaxis periods: gentamicin protocol plus ciprofloxacin (59 procedures), gentamicin-free protocol plus ciprofloxacin (43 procedures), and gentamicin-free protocol plus cefuroxime axetil (71 procedures). Because oral-antibiotic choice was nested within calendar time and changed only during the gentamicin-free era, it was treated as a time-varying co-intervention and was not modelled as an independently estimable treatment effect.

A preoperative urine culture without clinically significant bacterial growth was required. Mixed flora at ≤10^3^ colony-forming units/mL were accepted as contamination. Hair was clipped, not shaved, on the day before surgery. Initial skin cleansing was performed with a locally prepared aqueous mixture containing Manusan^®^ (4% chlorhexidine digluconate), Betadine^®^ (povidone–iodine) and 0.9% sodium chloride. A Betadine-containing aqueous solution was also used intraoperatively to irrigate the operative field and both wounds during surgery in both study periods. The proportions and final concentrations of the initial skin-preparation mixture, and the exact composition, volume and timing of the intraoperative Betadine-containing irrigation, were not retained in the source records. Routine preoperative methicillin-resistant *Staphylococcus aureus* screening was not performed, and vancomycin was not added to the prophylactic regimen in either period.

### 2.3. Outcomes and Follow-Up

Documented 30- and 90-day follow-up information was available for all procedures, with no loss to documented institutional follow-up. Longitudinal clinical follow-up ranged from 3 months to approximately 12 years, but comparative analyses were restricted to fixed 30-day or 90-day windows. The 90-day window was selected pragmatically to capture clinically apparent early events potentially related to intraoperative contamination; later presentation of an implantation-related infection remains biologically possible. Events occurring after day 90 were excluded from between-group inference because follow-up duration differed substantially between calendar periods.

For device-related outcomes, the dedicated database fields for surgical-site infection and urethral erosion were re-adjudicated by the principal investigator (M.A.S.) against the source clinical records and the timing of complete device removal. A clinician-recorded surgical-site infection (SSI) was defined as an infection diagnosed by the treating clinician as involving either operative wound or the implanted field after AMS 800 implantation and managed with systemic antibiotics and/or complete device removal; an isolated urinary tract infection was not included. The retrospective records did not uniformly retain the item-level criteria required to subclassify every SSI as superficial incisional, deep incisional or definite device infection; the qualified term clinician-recorded SSI is therefore used. The exact postoperative day of the gentamicin-protocol SSI could not be reconstructed. However, the same procedure had already met the 30-day all-cause endpoint because of a postoperative haematoma documented during the index hospitalisation; therefore, uncertainty in SSI timing did not affect the 30-day all-cause event count. This SSI was included unequivocally in the 90-day SSI outcome. In the gentamicin-free period, one SSI accompanied an erosion requiring explantation within 30 days and yielded extended-spectrum beta-lactamase (ESBL)-producing *Escherichia coli* from a wound culture obtained at explantation; the other involved the pressure-regulating balloon site on postoperative day 36, resolved after systemic antibiotic treatment and did not require explantation. Urethral erosion was defined by dated clinical or operative documentation. Complete explantation within 90 days for SSI and/or erosion was a retrospectively defined exploratory device-related clinical endpoint. It was not interpreted as an antimicrobial efficacy endpoint because urethral erosion commonly has a noninfectious mechanism. Documented 90-day SSI, erosion and explantation status was available for all procedures.

The broader safety outcomes were any postoperative complication documented within 30 days and within 90 days. SSI or erosion within either window was included in the corresponding all-cause outcome when its timing was classifiable. A restricted sensitivity outcome excluded urinary retention, voiding difficulty and prolonged catheterisation when these were the only recorded events. Early events were identified from inpatient, outpatient, emergency and operative documentation; externally managed events were included when recorded in the institutional follow-up record. Minor events managed exclusively outside the institution and never communicated to the study centre may not have been captured. Formal Clavien–Dindo grades were not uniformly available under the final endpoint definitions and were therefore not tabulated or used for variable selection or inferential modelling [14,15].

### 2.4. Statistical Analysis

Continuous variables were summarised as medians and interquartile ranges (IQRs) and compared with the Mann–Whitney U test. Categorical baseline variables were summarised as counts and percentages and compared with Fisher exact tests; the Fisher–Freeman–Halton exact test was used for the 2 × 3 procedure-type and pressure-regulating-balloon comparisons. Fixed 90-day clinician-recorded SSI, urethral erosion and complete explantation outcomes were reported as *n*/*N* (%), with absolute risk differences defined as the gentamicin-free rate minus the gentamicin-protocol rate. Two-sided 95% confidence intervals for risk differences were calculated with the Newcombe method based on Wilson score intervals, and Fisher exact tests were used for descriptive between-group comparisons. Because device-related events were sparse, no multivariable model was fitted for SSI, erosion or explantation. No inferential comparison was performed for events occurring after day 90.

For the broader all-cause 30-day and 90-day outcomes, crude odds ratios (ORs), absolute risk differences and Fisher exact *p* values were calculated. Exploratory association models were retained for each outcome, with gentamicin-free status forced into both models. Under the constraints imposed by 30 and 41 outcome events, the remaining covariates were selected post hoc on the basis of clinical interpretability and observed univariable associations; selection was therefore partly outcome informed. The 30-day model included reimplantation after previous explantation and intraoperative complication, whereas the 90-day model included reimplantation and prior endoscopic treatment for urethral stricture or VUAS. Intraoperative complication is a perioperative variable rather than a baseline confounder and may lie on a causal pathway; consequently, the models are not interpreted as causal adjustment models or estimates of an independent gentamicin effect. Penalised logistic regression, patient-cluster-robust standard errors, restriction to the first eligible procedure per patient, and complete-case models adding BMI and cardiovascular disease were used as sensitivity analyses and are summarised in Supplementary Table S1. BMI was missing in 19 procedures and was not imputed.

All eligible procedures were included, and no a priori sample-size calculation determined cohort size. With only three clinician-recorded SSIs and five unique 90-day complete explantations, the device-related analyses were exploratory and were not designed to establish equivalence or non-inferiority. No post hoc power calculation was performed; interpretation emphasised observed event counts and confidence intervals. Analyses were performed using IBM SPSS Statistics for Windows, version 31.0 (IBM Corp., Armonk, NY, USA). Patient-cluster-robust analyses were performed in Python 3.13 using statsmodels 0.14.6. Firth penalised logistic regression was performed using a custom Python implementation of Jeffreys-prior penalised likelihood; 95% confidence intervals and two-sided *p* values were Wald estimates based on the inverse numerical Hessian of the negative penalised log-likelihood. All tests were two-sided, and *p* < 0.05 was considered statistically significant.

## 3. Results

### 3.1. Cohort and Baseline Characteristics

**Figure 1:**
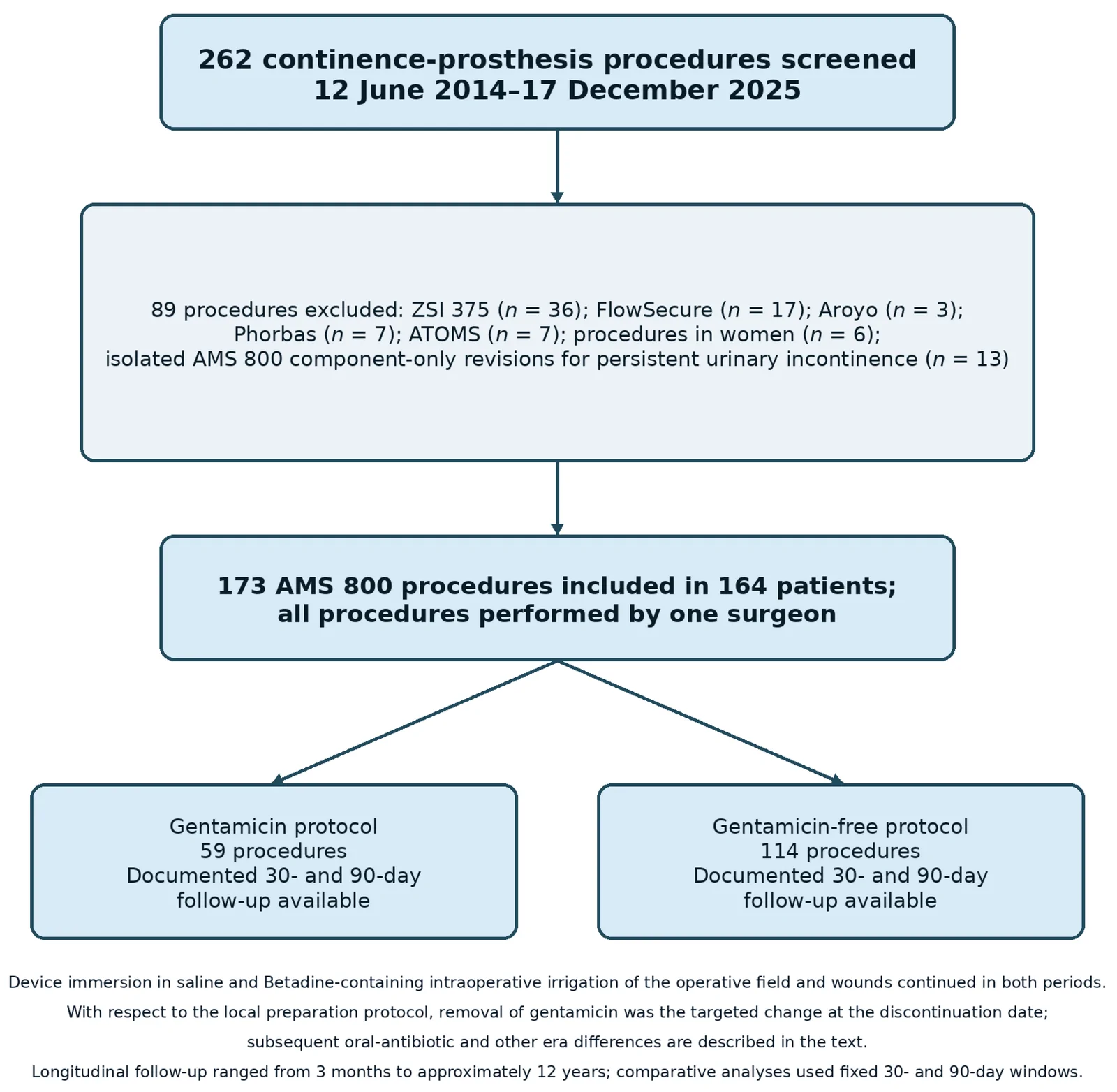
Flow of screened and included procedures. The 89 exclusions comprised 36 ZSI 375 implantations, 17 FlowSecure implantations, 3 Aroyo implantations, 7 Phorbas slings, 7 ATOMS slings, 6 procedures in women and 13 isolated component-only AMS 800 revisions for persistent urinary incontinence. Device immersion in saline and Betadine-containing intraoperative irrigation of the operative field and both wounds continued in both study periods. With respect to the local preparation protocol, removal of gentamicin was the targeted change at the discontinuation date; subsequent oral-antibiotic and other era differences are described in the text. Documented 30- and 90-day follow-up information was available for all included procedures. ATOMS, Adjustable Transobturator Male System; AUS, artificial urinary sphincter.

The final cohort included 173 AMS 800 procedures in 164 patients: 59 procedures (34.1%) under the gentamicin protocol and 114 (65.9%) under the gentamicin-free protocol. The procedure flow is shown in Figure 1. Documented 30- and 90-day follow-up information was available for all procedures. Baseline and procedural characteristics are summarised in Table 1.

Cardiovascular disease was more frequent and BMI was higher in the later gentamicin-free period. Operative time was substantially shorter in the gentamicin-free period (55 versus 76 min; *p* < 0.001), consistent with accumulated procedure-specific experience and more frequent concurrent preparation of the device by a resident in later years. Current smoking did not differ significantly. All 59 gentamicin-protocol procedures and the first 43 gentamicin-free procedures received post-discharge ciprofloxacin; the remaining 71 gentamicin-free procedures received cefuroxime axetil. These calendar-defined differences were treated as markers of era rather than independent treatment assignments.

### 3.2. Fixed 90-Day Surgical-Site Infection, Erosion and Explantation Outcomes

Within 90 days, clinician-recorded SSI occurred after 1/59 (1.7%) gentamicin-protocol procedures and 2/114 (1.8%) gentamicin-free procedures. The gentamicin-protocol SSI involved the operative field and led to complete device explantation; its exact postoperative day and organism could not be reconstructed. The same procedure had already met the 30-day all-cause endpoint because of a postoperative haematoma documented during the index hospitalisation. In the gentamicin-free group, one SSI accompanied an erosion requiring explantation within 30 days and yielded ESBL-producing *Escherichia coli* from a wound culture obtained at explantation; the other involved the pressure-regulating balloon site on postoperative day 36, resolved after systemic antibiotic treatment, and did not require explantation. Urethral erosion with complete explantation occurred after 0/59 gentamicin-protocol procedures and 4/114 (3.5%) gentamicin-free procedures. Thus, the retrospectively defined exploratory endpoint of complete explantation within 90 days for SSI and/or erosion occurred after 1/59 (1.7%) versus 4/114 (3.5%) procedures (risk difference +1.8 percentage points, 95% CI −5.8 to +7.2; *p* = 0.662). One erosion occurred within 30 days. No device-related multivariable regression was performed, and events after day 90 were not compared inferentially. Device-related outcomes are summarised in Table 2.

The ESBL-producing *E. coli* isolate was obtained from a wound culture at explantation in a case with clinical features of SSI and early urethral erosion. Gentamicin susceptibility was unavailable. Organism-level data were also unavailable for the gentamicin-protocol SSI and the postoperative day-36 lower-abdominal wound SSI.

### 3.3. All-Cause Early Outcomes and Complication Components

Thirty-day all-cause complications occurred in 14/59 (23.7%) gentamicin-protocol procedures and 16/114 (14.0%) gentamicin-free procedures. Ninety-day complications occurred in 15/59 (25.4%) and 26/114 (22.8%) procedures, respectively. Neither comparison was statistically significant, but the 95% CI for the 90-day risk difference remained compatible with an absolute excess of up to 10.0 percentage points after gentamicin withdrawal. In the restricted sensitivity analysis excluding retention, voiding difficulty and prolonged catheterisation when these were the only events, 30-day events occurred in 8/59 versus 11/114 procedures and 90-day events in 8/59 versus 19/114; the interpretation was unchanged. Broad all-cause outcomes and their clinical components are shown in Table 3 and Table 4.

### 3.4. Era, AMS 800 Selection and Oral-Antibiotic Subperiods

Accrual increased from 59 AMS 800 procedures over approximately 7.6 years in the gentamicin-protocol period to 114 over approximately 3.9 years in the gentamicin-free period. Lower AMS 800 counts in some earlier years coincided with greater use of alternative continence devices and therefore do not represent lower total prosthetic volume. The three calendar-defined prophylaxis periods comprised gentamicin protocol plus ciprofloxacin (*n* = 59), gentamicin-free protocol plus ciprofloxacin (*n* = 43), and gentamicin-free protocol plus cefuroxime axetil (*n* = 71); their 90-day all-cause event counts were 15/59, 6/43 and 20/71, respectively. These counts are descriptive only because oral-antibiotic choice, AMS 800 selection, operative experience and calendar time changed together.

### 3.5. Exploratory Association Models and Sensitivity Analyses

Exploratory association models were restricted to the broad all-cause outcomes and were not used for the sparse SSI, erosion or complete explantation endpoints. In the 30-day model, the OR for the gentamicin-free protocol was 0.50 (95% CI 0.22–1.15; *p* = 0.101); the model also included reimplantation after previous explantation and intraoperative complication. In the 90-day model, the OR for the gentamicin-free protocol was 0.86 (95% CI 0.40–1.84; *p* = 0.697); the model also included reimplantation and prior endoscopic treatment for urethral stricture or VUAS. Because covariate selection was partly outcome-informed and the 30-day model included a perioperative variable, these models are presented only as exploratory associations and not as causal estimates of an independent gentamicin effect. Patient-cluster-robust estimates were 0.50 (95% CI 0.21–1.18; *p* = 0.113) and 0.86 (95% CI 0.39–1.89; *p* = 0.707), and first-eligible-procedure estimates were 0.46 (95% CI 0.20–1.08; *p* = 0.074) and 0.82 (95% CI 0.38–1.77; *p* = 0.608), for the 30- and 90-day outcomes, respectively. Firth and complete-case estimates are shown in Supplementary Table S1. All confidence intervals were wide and compatible with both benefit and harm.

## 4. Discussion

### 4.1. Principal Findings

After local gentamicin was withdrawn, no statistically significant between-period difference was detected in clinician-recorded SSI, the retrospectively defined exploratory 90-day complete explantation endpoint, or the broader all-cause 30-day and 90-day outcomes. Complete explantation within 90 days for SSI and/or urethral erosion occurred after 1/59 (1.7%) gentamicin-protocol procedures and 4/114 (3.5%) gentamicin-free procedures. The confidence interval for the absolute difference was wide and included both benefit and harm. These data do not show equivalence and do not prove that local gentamicin lacks benefit.

The infection-only outcome and the device-related clinical composite require separate interpretation. One clinician-recorded SSI occurred in the gentamicin-protocol group and two occurred in the gentamicin-free group. One gentamicin-free SSI accompanied an early erosion, and ESBL-producing *E. coli* was cultured from the wound at explantation; the other was a postoperative day-36 SSI at the pressure-regulating balloon site that resolved without explantation. Four urethral erosions occurred in the gentamicin-free group, and all led to complete device removal. Although local gentamicin could theoretically influence bacterial contamination when infection coexists with erosion, urethral erosion is not primarily an infection-mediated endpoint: early erosion more often reflects unrecognised urethral injury, impaired perfusion or cuff-related pressure, while infection may coexist with an established erosion [1]. The composite was therefore used as a clinically consequential, retrospectively defined exploratory device-removal endpoint, not as a direct measure of antimicrobial efficacy. The very small number of events means that a clinically relevant difference cannot be excluded.

The broad all-cause outcomes increased event capture but were dominated by retention, catheterisation and bleeding—events not expected to be prevented by local antibiotics. They are useful as general early safety outcomes but are not direct measures of antimicrobial efficacy. The restricted sensitivity analysis did not materially change the interpretation. Accordingly, the data support only the conclusion that no statistically significant between-period difference was detected in the observed early outcomes; they do not demonstrate that local gentamicin is ineffective.

### 4.2. Era Effects, AMS 800 Selection and the Single-Surgeon Design

All procedures were performed by the same surgeon, eliminating changes in surgeon composition but not temporal confounding within that surgeon’s practice. AMS 800-specific experience increased, operative time fell from a median of 76 to 55 min, and a resident more often prepared the device concurrently in later years. In addition, alternative continence devices were selected more often during parts of the earlier period, so the patients receiving AMS 800 may not have represented an identical clinical spectrum throughout the study. Referral patterns, case mix, team organisation and documentation may also have evolved.

Gentamicin exposure was assigned entirely by calendar period, so protocol status remained inseparable from other era-related changes in this before–after design. The change from ciprofloxacin to cefuroxime axetil created a third prophylaxis period nested within the gentamicin-free era. Descriptive subperiod counts cannot separate local gentamicin from oral antibiotic choice, AMS 800 selection, accumulated experience or other secular changes. The study should therefore be interpreted as a pragmatic stop-and-verify evaluation of one de-implemented step, not as a causal comparison of otherwise exchangeable groups.

### 4.3. Local Irrigation Evidence and Relevance of Other Implant Fields

The literature requires separation of three different interventions: incisional wound irrigation, irrigation of an implant pocket and soaking or rinsing of the prosthesis itself. WHO guidance against antibiotic incisional irrigation does not directly answer whether an entire prosthetic device should be immersed in an antibiotic solution. Conversely, device-dipping data do not establish the value of irrigating two operative wounds. In the gentamicin-protocol period, the complete uncoated AMS 800 system was immersed in gentamicin-containing saline, and both wounds were irrigated with the same solution. In the gentamicin-free period, device immersion in 0.9% sodium chloride and Betadine-containing intraoperative irrigation of the operative field and both wounds continued, but local gentamicin was omitted. The study therefore compares a gentamicin-containing with a gentamicin-free local preparation protocol and cannot separate any effect of gentamicin device immersion from that of gentamicin wound irrigation.

Penile-prosthesis observations are closer to AUS surgery but remain device-specific. An inflatable penile prosthesis has a different geometry and a larger implanted surface than an AMS 800 system, which limits direct extrapolation of dipping results. Multicentre studies show that guideline-concordant or broader antimicrobial exposure does not automatically translate into lower prosthetic infection rates [16,17]. In a retrospective multicentre study of diabetic men receiving hydrophilic-coated penile implants, vancomycin-plus-gentamicin dipping was associated with fewer infections than other dipping solutions [18]. These studies support equipoise rather than direct extrapolation to uncoated AMS 800 devices.

The stewardship rationale remains relevant. Local antibiotic exposure can be poorly standardised and may escape routine stewardship monitoring. Pharmacokinetic data, historical reports of gentamicin-resistant organisms after topical exposure and AUS biofilm studies support caution with unproven gentamicin use, although they do not prove that a single intraoperative rinse selects resistance [19,20,21].

Manufacturer documentation also requires nuance. AMS 800 operating-room documentation has listed antibiotic solution among possible operative supplies, but a supply or handling instruction is not comparative efficacy evidence. The 2023 Boston Scientific prescriptive information states that InhibiZone components should not be soaked in saline or other solutions and may only be briefly rinsed or dipped in a sterile solution immediately before implantation [22]. All devices in this cohort were uncoated, so this InhibiZone-specific restriction did not apply directly; nevertheless, it illustrates why precise descriptions of coating and component handling are essential.

Evidence from orthopaedic, cardiac-device and breast-implant surgery was considered only as indirect context. Trials and reviews in these fields have produced mixed findings for povidone-iodine, antibiotic pocket irrigation and multicomponent prophylactic bundles [23,24,25,26]. Differences in implant material, anatomy, microbial ecology, solution, contact time and outcome definition prevent direct extrapolation to uncoated AMS 800 devices. These data therefore reinforce the need for procedure-specific evidence rather than support a particular AUS irrigation solution.

Because device immersion in 0.9% sodium chloride and Betadine-containing intraoperative irrigation of the operative field and both wounds continued after gentamicin withdrawal, the study does not compare local preparation with no local preparation. This continuity reduces confounding by complete cessation of device immersion or antiseptic use, but gentamicin device immersion and gentamicin wound irrigation were withdrawn together and their individual contributions cannot be separated.

### 4.4. Exploratory Association Models

Exploratory models were included only to examine the robustness of the association between local protocol status and broad all-cause outcomes. Because covariate selection was partly outcome-informed, exposed subgroups were small, and the outcomes were heterogeneous, coefficients for the adjustment variables should not be interpreted as independent clinical predictors.

### 4.5. Systemic and Post-Discharge Prophylaxis

The one-day ceftriaxone–amikacin–metronidazole regimen was stable and developed with the hospital microbiologist, but its antimicrobial spectrum is broader than many guideline schemes [10,27]. It already includes an aminoglycoside, so local gentamicin was an additional exposure layered onto broad systemic prophylaxis. The present results must not be used to advocate broad intravenous coverage; prospective evaluation of a narrower microbiology-informed regimen remains an important stewardship question.

All patients also received 10 days of oral antibiotics after discharge. Ciprofloxacin was used through May 2023 and cefuroxime axetil thereafter following European fluoroquinolone restrictions [13]. This later change was completely linked to calendar time and occurred only in the gentamicin-free era. The current data cannot distinguish the effects of local gentamicin, broad intravenous prophylaxis, prolonged oral exposure or the oral-drug switch. Accordingly, the study supports only a centre-specific assessment of gentamicin withdrawal on top of this regimen and cannot be generalised to centres using shorter or narrower prophylaxis.

### 4.6. Limitations

This study was retrospective, single-centre and non-randomised. Gentamicin exposure was defined by a single calendar date, making it inseparable from era. Although one surgeon performed all procedures, increasing AMS 800-specific experience, shorter operative time, more frequent resident preparation of the device, evolving referral patterns and possible changes in selection of AMS 800 rather than alternative devices remain potential confounders. The later switch from ciprofloxacin to cefuroxime axetil was an additional time-varying co-intervention. Descriptive subperiod comparisons cannot remove these limitations.

The number of device-related events was very small: three clinician-recorded SSIs, four erosions and five unique complete explantations within 90 days. The retrospectively defined complete explantation endpoint combined events with different mechanisms and was therefore exploratory rather than an antimicrobial efficacy endpoint. The exact postoperative day of the gentamicin-protocol SSI could not be reconstructed, preventing a separate fixed 30-day SSI comparison, although this uncertainty did not affect the 30-day all-cause event count because the same procedure had a haematoma during the index hospitalisation. The wide confidence intervals cannot exclude clinically important benefit or harm and preclude equivalence or non-inferiority claims. The broader all-cause outcomes also combined events with different mechanisms. Outcome re-adjudication was performed by a single investigator who was aware of the calendar period, although erosion and explantation were verified against dated clinical and operative records.

Documented 30- and 90-day follow-up information was available for all procedures, with no loss to documented institutional follow-up. Ascertainment relied on retrospective institutional records; minor events managed exclusively outside the institution and never communicated to the centre may not have been captured. Longitudinal follow-up ranged from 3 months to approximately 12 years, but events after day 90 were deliberately excluded from comparative inference because follow-up was unequal between eras. The 90-day window was pragmatic and does not exclude later presentation of infection related to implantation. Culture and susceptibility information was incomplete, and gentamicin susceptibility was unavailable for the ESBL-producing *E. coli* isolate.

BMI was missing in 19 procedures, preoperative pad data were incomplete, and American Society of Anesthesiologists physical status class and glycated haemoglobin (HbA1c) were unavailable. Repeated procedures created potential within-patient dependence, although the numerical patient-cluster-robust and first-procedure estimates shown in Supplementary Table S1 did not materially change the interpretation. Covariate selection for the exploratory models was partly outcome-informed, and intraoperative complication was not a baseline confounder. All devices were uncoated, which improves internal consistency but limits extrapolation to InhibiZone-coated systems. Device immersion in saline and Betadine-containing intraoperative irrigation of the operative field and both wounds were common to both periods, but gentamicin device immersion and gentamicin wound irrigation were withdrawn together; their individual effects cannot be separated. Most importantly, the broad intravenous regimen and universal 10-day oral course substantially limit generalisability to other prophylaxis strategies.

## 5. Conclusions

Within this single-centre cohort, no statistically significant between-period difference was detected in 90-day clinician-recorded SSI, the exploratory endpoint of complete explantation for SSI and/or urethral erosion, or broad all-cause 30- and 90-day complications after local gentamicin was withdrawn. However, device-related events were sparse, and gentamicin exposure was inseparable from calendar time and other changes in perioperative practice. These findings do not establish equivalence and do not exclude a clinically relevant benefit or harm from local gentamicin.

## Figures and Tables

**Table 1:** Baseline and procedural characteristics by local gentamicin exposure.

Characteristic	Gentamicin Protocol (*n* = 59)	Gentamicin-Free Protocol (*n* = 114)	*p* Value
Age, years	69.0 [63.5–73.5]	68.5 [63.0–72.0]	0.778
BMI, kg/m^2^	25.8 [24.4–28.2] (*n* = 51)	28.4 [25.5–31.1] (*n* = 103)	0.007
Current smoker	5/59 (8.5%)	3/114 (2.6%)	0.124
Cardiovascular disease	10/59 (16.9%)	43/114 (37.7%)	0.005
Diabetes	7/59 (11.9%)	25/114 (21.9%)	0.148
Antiplatelet therapy	7/59 (11.9%)	14/114 (12.3%)	1.000
Anticoagulant therapy	6/59 (10.2%)	5/114 (4.4%)	0.188
Radical prostatectomy	48/59 (81.4%)	93/114 (81.6%)	1.000
Prior endoscopic treatment for urethral stricture or VUAS	15/59 (25.4%)	37/114 (32.5%)	0.385
Previous urethroplasty	7/59 (11.9%)	17/114 (14.9%)	0.649
Pelvic radiotherapy	25/59 (42.4%)	52/114 (45.6%)	0.748
Procedure type: primary/complete device exchange/reimplantation after explantation	43 (72.9%)/11 (18.6%)/5 (8.5%)	92 (80.7%)/16 (14.0%)/6 (5.3%)	0.462
Cuff placement: standard bulbar/transcorporal	50 (84.7%)/9 (15.3%)	101 (88.6%)/13 (11.4%)	0.479
Cuff size, cm	4.5 [4.0–4.5]	4.5 [4.0–5.0] (*n* = 113)	0.069
Pressure-regulating balloon range: 51–60/61–70/71–80 cm H_2_O	2/56/1	0/109/3 (*n* = 112)	0.176
Operative time, min	76 [65–90]	55 [55–60]	<0.001
Length of stay, days	2 [2–3]	3 [2–3] (*n* = 112)	0.004
Catheter duration, h	20 [20–20]	20 [20–20]	0.054

Continuous variables are median [IQR]; categorical variables are *n*/*N* (%) unless otherwise specified. Current smoker denotes smoking at the time of surgery; former smoking was not systematically recorded. Fisher–Freeman–Halton tests were used for the 2 × 3 procedure-type and pressure-regulating-balloon comparisons. BMI, body mass index; IQR, interquartile range; VUAS, vesicourethral anastomotic stenosis.

**Table 2:** Fixed 90-day surgical-site infection, erosion and complete explantation outcomes by local gentamicin exposure.

Outcome	Gentamicin Protocol (*n* = 59)	Gentamicin-Free Protocol (*n* = 114)	Risk Difference, pp (95% CI)	*p* Value
Clinician-recorded surgical-site infection ≤ 90 days	1/59 (1.7%)	2/114 (1.8%)	+0.1 (−7.4 to +4.7)	1.000
Urethral erosion with complete explantation ≤ 90 days	0/59 (0%)	4/114 (3.5%)	+3.5 (−3.0 to +8.7)	0.301
Any complete explantation for surgical-site infection and/or erosion ≤ 90 days	1/59 (1.7%)	4/114 (3.5%)	+1.8 (−5.8 to +7.2)	0.662

Clinician-recorded SSI and complete explantation were adjudicated as separate outcomes. The ESBL-producing *E. coli* case was classified as both SSI and erosion but contributed only once to the complete explantation endpoint. All four early erosions led to complete explantation. The complete explantation composite was retrospectively defined as an exploratory device-related clinical endpoint and was not interpreted as an antimicrobial efficacy endpoint because erosion may arise through noninfectious mechanisms. Risk differences are gentamicin-free minus gentamicin-protocol rates; 95% CIs were calculated with the Newcombe method. Fisher exact tests were used. No comparison of events after day 90 was performed.

**Table 3:** Broad all-cause early outcomes by local gentamicin exposure.

Endpoint	Gentamicin Protocol (*n* = 59)	Gentamicin-Free Protocol (*n* = 114)	Risk Difference, pp (95% CI)	OR (95% CI)	*p* Value
30-day all-cause endpoint	14/59 (23.7%)	16/114 (14.0%)	−9.7 (−23.0 to +2.1)	0.52 (0.24–1.17)	0.138
90-day all-cause endpoint	15/59 (25.4%)	26/114 (22.8%)	−2.6 (−16.7 to +10.0)	0.87 (0.42–1.80)	0.710

Risk differences are gentamicin-free minus gentamicin-protocol rates and are expressed in percentage points (pp), with two-sided 95% CIs. ORs compare the gentamicin-free protocol with the gentamicin protocol. OR, odds ratio.

**Table 4:** Clinical components of early complications by local gentamicin exposure using full group denominators.

Clinical Component	30 Days: Gentamicin	30 Days: Gentamicin-Free	90 Days: Gentamicin	90 Days: Gentamicin-Free
Urinary retention or voiding difficulty requiring catheterisation or drainage	10/59 (16.9%)	8/114 (7.0%)	11/59 (18.6%)	9/114 (7.9%)
– Prolonged catheterisation (>1 day)	10/59 (16.9%)	7/114 (6.1%)	10/59 (16.9%)	7/114 (6.1%)
– Urinary retention requiring cystostomy/suprapubic catheter	1/59 (1.7%)	4/114 (3.5%)	2/59 (3.4%)	5/114 (4.4%)
– Single catheterisation for voiding difficulty	0/59 (0%)	1/114 (0.9%)	0/59 (0%)	1/114 (0.9%)
Haematoma or bleeding	8/59 (13.6%)	8/114 (7.0%)	8/59 (13.6%)	8/114 (7.0%)
– Reoperation for bleeding at the pressure-regulating balloon site	0/59 (0%)	1/114 (0.9%)	0/59 (0%)	1/114 (0.9%)
Clinician-recorded surgical-site infection	Not classifiable *	1/114 (0.9%)	1/59 (1.7%)	2/114 (1.8%)
Urethral erosion	0/59 (0%)	1/114 (0.9%)	0/59 (0%)	4/114 (3.5%)
Device-related reintervention for malfunction, cuff/pump revision, or pump malposition	1/59 (1.7%)	0/114 (0%)	1/59 (1.7%)	3/114 (2.6%)
Urinary tract infection without SSI	0/59 (0%)	1/114 (0.9%)	0/59 (0%)	1/114 (0.9%)
Bladder stone requiring cystolithotripsy	0/59 (0%)	0/114 (0%)	0/59 (0%)	1/114 (0.9%)

Components are descriptive and are not mutually exclusive; rows beginning with an en dash are subcategories of the immediately preceding parent category. Therefore, component counts can exceed the number of procedures meeting an all-cause endpoint. * The exact day of the gentamicin-protocol SSI was not reconstructable, so it is not assigned to the 30-day SSI component; the same procedure nevertheless met the 30-day all-cause endpoint because of a haematoma during the index hospitalisation. UTI, urinary tract infection.

## Data Availability

The data analysed during the current study are not publicly available because of patient privacy and institutional restrictions but may be available from the corresponding author on reasonable request and subject to applicable ethics approval and data-sharing regulations.

## Supplementary Materials

The following supporting information can be downloaded at: https://www.mdpi.com/article/10.3390/antibiotics15090894/s1, Table S1: Sensitivity estimates for the association between the gentamicin-free protocol and broad all-cause complications.

## Abbreviations

AUS, artificial urinary sphincter; BMI, body mass index; CI, confidence interval; ESBL, extended-spectrum beta-lactamase; HbA1c, glycated haemoglobin; IQR, interquartile range; OR, odds ratio; pp, percentage points; SSI, surgical-site infection; UTI, urinary tract infection; VUAS, vesicourethral anastomotic stenosis; WHO, World Health Organization.
