# Supplementary material for: Early Outcomes After Discontinuing Routine Gentamicin Irrigation During AMS 800 Artificial Urinary Sphincter Implantation: A Single-Centre Retrospective Before–After Cohort Study
Source: Antibiotics (Basel). 2026 Sep 11;15(9):894. doi: 10.3390/antibiotics15090894 (PMC13603501; doi:10.3390/antibiotics15090894)
Supplement: Supplementary file 1 [file antibiotics-15-00894-s001.zip › antibiotics-4526988-supplementary.pdf]

Supplementary Material

Early outcomes after discontinuing routine gentamicin irrigation during AMS 800 artificial urinary sphincter implantation

Manuscript ID: antibiotics-4526988

Supplementary Table S1. Sensitivity estimates for the association between the gentamicin-free protocol and broad all-cause complications.

Analysis	30-day OR (95% CI; p)	90-day OR (95% CI; p)
Standard maximum-likelihood model	0.50 (0.22–1.15; 0.101)	0.86 (0.40–1.84; 0.697)
Firth penalised logistic regression	0.51 (0.22–1.15; 0.104)	0.86 (0.40–1.82; 0.687)
Patient-cluster-robust standard errors	0.50 (0.21–1.18; 0.113)	0.86 (0.39–1.89; 0.707)
First eligible procedure per patient	0.46 (0.20–1.08; 0.074)	0.82 (0.38–1.77; 0.608)
Complete-case model adding BMI and cardiovascular disease	0.41 (0.16–1.03; 0.057)	0.82 (0.36–1.88; 0.635)

ORs compare the gentamicin-free protocol with the gentamicin protocol. Core covariate sets were those described in Section 2.4: the 30-day model included reimplantation after previous explantation and intraoperative complication, and the 90-day model included reimplantation and prior endoscopic treatment for urethral stricture or VUAS. Only the protocol coefficient is presented because these exploratory models were used solely to examine the robustness of the protocol association; coefficients for the adjustment variables were not interpreted as independent clinical predictors. The patient-cluster-robust analysis retained all procedures and adjusted standard errors for repeated procedures within patients. The first-procedure analysis included the first eligible procedure for each of 164 patients. Complete-case models adding BMI and cardiovascular disease included 154 procedures (27 and 37 events at 30 and 90 days); BMI was missing in 19 procedures and was not imputed. Firth penalised logistic regression used a custom Python implementation of Jeffreys-prior penalised likelihood; confidence intervals and p values were Wald estimates based on the inverse numerical Hessian of the negative penalised log-likelihood. BMI, body mass index; CI, confidence interval; OR, odds ratio; VUAS, vesicourethral anastomotic stenosis.
